# The complex nature of calcium cation interactions with phospholipid bilayers

**DOI:** 10.1038/srep38035

**Published:** 2016-12-01

**Authors:** Adéla Melcrová, Sarka Pokorna, Saranya Pullanchery, Miriam Kohagen, Piotr Jurkiewicz, Martin Hof, Pavel Jungwirth, Paul S. Cremer, Lukasz Cwiklik

**Affiliations:** 1 J. Heyrovský Institute of Physical Chemistry, Academy of Sciences of the Czech Republic, v.v.i., Dolejškova 3, Prague, 18223, Czech Republic; 2 Department of Chemistry, Pennsylvania State University, University Park, PA 16802, United States; 3 Institute of Organic Chemistry and Biochemistry, Academy of Sciences of the Czech Republic, Flemingovo nám. 2, Prague, 16610, Czech Republic; 4 Institute for Computational Physics, University of Stuttgart, Allmandring 3, Stuttgart, 70569, Germany; 5 Department of Physics, Tampere University of Technology, POB 692, Tampere, FI-33101, Finland; 6 Department of Biochemistry and Molecular Biology, Pennsylvania State University, University Park, PA 16802, United States

## Abstract

Understanding interactions of calcium with lipid membranes at the molecular level is of great importance in light of their involvement in calcium signaling, association of proteins with cellular membranes, and membrane fusion. We quantify these interactions in detail by employing a combination of spectroscopic methods with atomistic molecular dynamics simulations. Namely, time-resolved fluorescent spectroscopy of lipid vesicles and vibrational sum frequency spectroscopy of lipid monolayers are used to characterize local binding sites of calcium in zwitterionic and anionic model lipid assemblies, while dynamic light scattering and zeta potential measurements are employed for macroscopic characterization of lipid vesicles in calcium-containing environments. To gain additional atomic-level information, the experiments are complemented by molecular simulations that utilize an accurate force field for calcium ions with scaled charges effectively accounting for electronic polarization effects. We demonstrate that lipid membranes have substantial calcium-binding capacity, with several types of binding sites present. Significantly, the binding mode depends on calcium concentration with important implications for calcium buffering, synaptic plasticity, and protein-membrane association.

Calcium cations are very potent and versatile agents within a cell. Their divalent character makes them strong binders; hence, their physiological concentration must be strictly controlled. The concentration of Ca^2+^ in the extracellular space is about 2 mM, while its intracellular levels are much lower and range amongst cell organelles from 100 nM in the cytosol to 600 μM in the endoplasmic reticulum^1^,^2^. The maintenance of low intracellular calcium concentrations is achieved, among other mechanisms, by calcium buffers which include both cytoplasmic and membrane-anchored calcium-binding proteins^1^,^3^,^4^. On the other hand, key events in calcium signaling are connected with rapid spikes of Ca^2+^ concentration caused by an influx of the Ca^2+^ into the cytosol via calcium channels^5^. These spikes can be up to 100-fold higher compared to the resting Ca^2+^ concentration and are both spatially and temporally modulated by the calcium buffers^4^.

There is one commonly overlooked factor that significantly influences behavior of calcium ions upon their influx into the cytosol. Namely, apart from protein-based buffers, calcium ions strongly interact with the negatively charged inner leaflet of the plasma membrane^6^. Recently, it has been shown that adsorption of calcium can neutralize this negative charge, and that this effect alone is responsible for the modulation of T-cell activation^7^. Calcium ions can also serve as a bridge between a protein and the cell membrane, for instance, during membrane association of C2 domains and annexins^8^,^9^. Ca^2+^-membrane binding is also recognized as a key factor during membrane fusion^10^.

The interactions of calcium ions with lipid membranes have been probed by a variety of experimental methods^11^,^12^,^13^,^14^. It is generally accepted that the presence of Ca^2+^ rigidifies and orders lipid bilayers^13^,^15^,^16^,^17^,^18^,^19^. Conformational changes of the lipid headgroup region^20^,^21^, ordering of acyl chains^20^,^21^,^22^, and lipid dehydration^17^,^23^,^24^,^25^,^26^ were reported.

Since physiological concentrations of calcium are low and calcium domains are highly localized in space, the global membrane changes induced by its binding are highly relevant as well as the identity of the local binding sites. Lipid bilayers consisting of 80 mol% phosphatidylcholine (PC) and 20 mol% phosphatidylserine (PS) have often been used as a simple model of the inner leaflet of the plasma membrane. In this system, three possible binding sites can be distinguished: carboxyl groups of PS, phosphate groups of PC and PS, and carbonyl groups of PC and PS. Experimental methods, predominantly NMR-based, identified two distinct binding modes of calcium to PC/PS membrane, but their nature is not completely resolved^20^,^27^. Many studies demonstrated that calcium binds primarily to phosphate groups of all phospholipids, independent of their charge^28^, even in pure PC membranes^17^,^29^,^30^. Molecular dynamics simulations have provided more details and generally confirmed binding to phosphate groups in PC and several anionic lipids, including PS^18^,^26^,^31^,^32^, but concurrent binding to the carboxyl group of PS has also been reported^18^,^31^. It was suggested that calcium can also bind to the carbonyl oxygen^25^,^33^, which is in line with the binding of Na^+^ and K^+^ to PC and PC/PS carbonyls^34^. Regarding the local consequences of calcium binding, simulations indicate that Ca^2+^ is able to cluster phospholipid molecules via ion-bridges^18^,^26^,^32^.

Despite previous studies, an atomic-level understanding of calcium-membrane interactions is still incomplete and often inconsistent. In particular, earlier MD studies suffered from very short (<100 ns) trajectories yielding unconverged results; but more importantly, the previously used force fields overshoot ion-lipid binding interactions^35^. In the present work we overcome these limitations by utilizing a more realistic ionic force field implicitly accounting for polarization effects. We combine MD simulations with experimental methods to gain a comprehensive complex molecular-level picture of calcium ion-lipid bilayer binding.

## Results

In the subsequent sections we present the results of experimental and computational investigations on the interactions of calcium ions with model zwitterionic and anionic lipid systems.

### Dynamic light scattering (DLS) and zeta potential measurements

**Figure 1:**
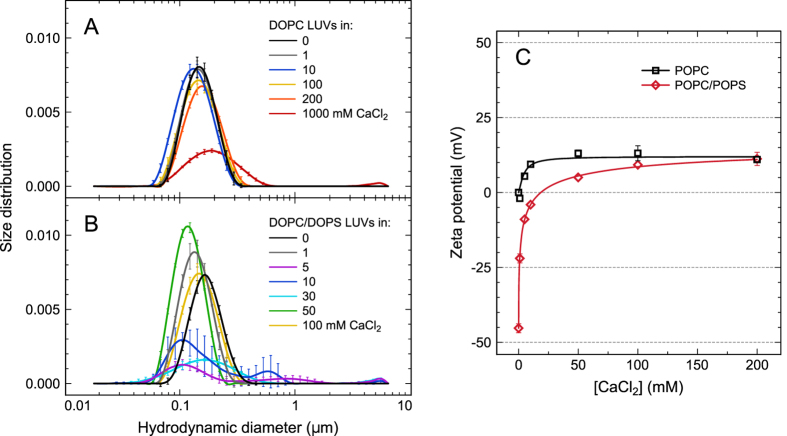
Liposome size distribution and zeta potential. (**A**,**B**) Hydrodynamic diameter of extruded large unilamellar vesicles composed of DOPC (**A**) and DOPC/DOPS (4:1, mol:mol) (**B**). Error bars represent SD, n ≥ 4. (**C**) Zeta potential of POPC and POPC/POPS (4:1, mol:mol) large unilamellar vesicles measured as a function of CaCl_2_ concentration. Error bars represent SD, n ≥ 3. Data fitted with Langmuir-Freundlich model.

First, we present the calcium induced changes of macroscopic properties of DOPC and DOPC/DOPS vesicles. During preparation of PC/PS vesicles, precipitation of lipid aggregates was observed in samples containing 5 to 30 mM CaCl_2_. It has been shown that calcium can lead to aggregation or fusion of negatively charged lipid vesicles^36^,^37^. To check whether these phenomena occurred in the present systems, DLS was used to measure the size distribution of DOPC and DOPC/DOPS (80/20, mol/mol) vesicles. Representative results are shown in Fig. 1. Unimodal distributions of liposome hydrodynamic diameters centered near 150 nm were obtained, as expected, for most of the DOPC samples (Fig. 1A). Vesicle aggregation was only induced at 1 M CaCl_2_ which was manifest in particle growth, distribution broadening and the appearance of very large (~5 μm) aggregates. The DOPC/DOPS vesicles were found to aggregate already at 5 to 30 mM CaCl_2_ (Fig. 1B). Samples became polydisperse with largest detectable particles being >5 μm. Interestingly at 50 to 1000 mM CaCl_2_, the samples were monodisperse again. Also the addition of CaCl_2_ to already aggregated samples reversed the aggregation. There were no indications of any persistent growth of the liposomes, which would be expected in the case of liposome fusion. This means that calcium ions are able to bridge the lipid bilayers of neighboring vesicles and that this process is reversible. This agrees with previous report on calcium-induced vescicle fusion (e.g. refs 12 and 24).

To further investigate vesicle clustering, the zeta potential of POPC and POPC/POPS vesicles was measured (Fig. 1C). The zeta potential of POPC vesicles, which in the absence of CaCl_2_ was close to zero, increased in the presence of calcium ions and stabilized at ~15 mV. This indicates the adsorption of ions. For POPC/POPS vesicles, the initial zeta potential of about −45 mV became positive above 25 mM CaCl_2_ and at 200 mM reached the same value as the POPC vesicles. This is direct evidence of overcharging of the originally negatively charged POPC/POPS vesicles by adsorbed calcium ions. These results rationalize the loss of DOPC/DOPS vesicle monodispersity at intermediate CaCl_2_ concentrations, while higher concentrations led to overcharging and, as a consequence, electrostatic repulsion of the subsequently positively charged vesicles. This implies that the driving force for the adsorption of calcium ions to PC/PS membranes goes beyond simple Coulomb interactions between permanent charges or dipoles. Overcharging has been previously observed in many colloidal systems including lipid membranes, peptides, proteins, and DNA (e.g. refs 38 and 39).

### Time-dependent fluorescence shift (TDFS) in PC and PC/PS bilayers

**Figure 2:**
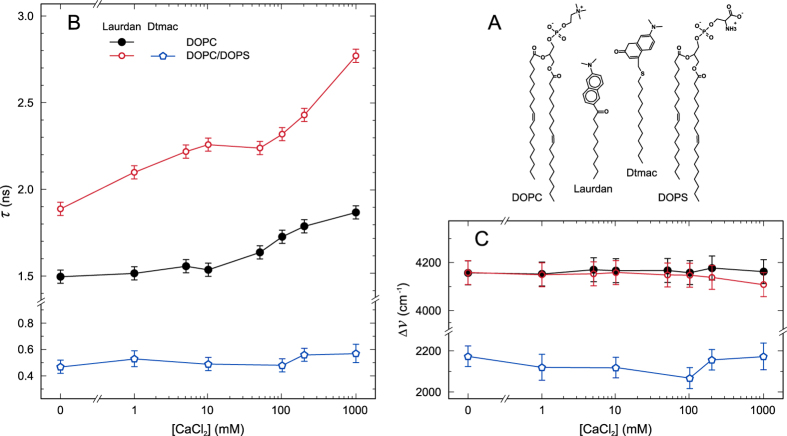
Laurdan and Dtmac Time Dependent Fluorescence Shift (TDFS). (**A**) Location of the probes with respect to DOPC and DOPS molecules in membranes. (**B**) Integrated relaxation time, τ, and (**C**) total spectral shift, Δν, measured for 1 mol% of the probes embedded in large unilamellar vesicles of various composition dispersed in water or in CaCl_2_ solutions of various concentrations (1–1000 mM). Measured at 283 K; error bars represent SD, n ≥ 2.

The above macroscopic experiments provided motivation for further molecular investigations of the calcium binding sites. Laurdan and Dtmac probes were used to investigate the consequences of calcium adsorption at the level of carbonyls and phosphates, respectively, of PC and PC/PS membranes (Fig. 2A). The fluorophore of Laurdan, located at ~10 Å from the DOPC bilayer center^40^,^41^, was shown to probe predominantly polarity and mobility of hydrated *sn*-1 carbonyls of phospholipids^42^. The fluorophore of Dtmac is located close to the phosphate moieties of lipid headgroups, i.e., ~15 Å from the DOPC bilayer center (A. Olżyńska, personal communication). TDFS parameters measured for dioleoylic lipid vesicles at 1 to 1000 mM CaCl_2_ are shown in Fig. 2. Comparison of DOPC/DOPS with POPC/POPS is given in the Supplementary Information. The influence of Cl^−^ ions on the membrane properties is minor, as was evidenced by TDFS and rationalized by MD in our previous studies^43^,^44^.

The Laurdan relaxation time (*τ*) shows that calcium restricts mobility of the carbonyls (Fig. 2B, red curves) of both neutral and negatively charged lipids. This effect is much stronger in the case of the negatively charged DOPC/DOPS vesicles, for which three different regions can be distinguished in the curve of *τ* as a function of CaCl_2_ concentration. First, the steep increase in the range of 1 to 5 mM CaCl_2_ demonstrates that even such a small amount of calcium can considerably reduce the mobility of lipid carbonyls (i.e. the influence of 1 mM CaCl_2_ is comparable with the one for 150 mM NaCl)^45^. Second, between 5 and 50 mM CaCl_2_, a plateau is observed where the initial effect of calcium seems saturated. In the third region, 50–1000 mM CaCl_2_, a second slowdown of the relaxation is present. In this region, the increase of *τ* is much less steep than in the first one (note that Fig. 2B presents a semi-logarithmic plot). The slopes in the first and the third regions are ~55 and ~0.5 ns/M, respectively. Overall, such a complex calcium concentration dependence suggests the existence of various interaction modes or diverse binding sites in the PC/PS bilayer. In the case of DOPC, no differences in Laurdan *τ* values were noted up to 10 mM CaCl_2_ (Fig. 2B, black curve). For 50 mM and more, CaCl_2_ gradually increased the relaxation time; its slope between 10 and 200 mM is ~1 ns/M.

The total spectral shift of Laurdan representing local polarity at the level of lipid carbonyls was not affected by calcium binding (Fig. 2C). A minor dehydration is visible only for 1 M CaCl_2_ in the DOPC/DOPS system. It was shown that the presence of an ion itself in the vicinity of the fluorescent probe can contribute to the relaxation process and increase Δ*ν*^46^, which could compensate for the effect of possible dehydration of the lipid carbonyls.

Dtmac responds to the increasing calcium concentration much less dramatically than Laurdan does. Due to the presence of bulk water in the vicinity of Dtmac, the relaxation is very fast with a considerable part being faster than the time resolution of our measurements. Herein, only the Dtmac data for negatively charged DOPC/DOPS vesicles are presented (Fig. 2, blue curves). It is very likely that the hydrated carboxylate moieties of DOPS molecules also contribute to the relaxation probed by Dtmac in a manner dependent on the PS headgroup orientation. The kinetics of the relaxation measured with Dtmac was only weakly affected at 0.2 and 1 M CaCl_2_. The total spectral shift was almost unaffected by the presence of calcium. A slight dehydration, observed in the range of 1–100 mM CaCl_2_, was reversed at 0.2 and 1 M CaCl_2_. The latter can again be interpreted as an indication of the close proximity of calcium ions to the Dtmac fluorophore. Overall, the above results indicate that calcium affects the headgroup region mobility at considerably higher concentrations than for the carbonyl region.

Although the plateau of *τ* observed for Laurdan in DOPC/DOPS at 5 to 50 mM CaCl_2_ coincides with aggregation of vesicles at 5 to 30 mM CaCl_2_ reported by DLS, we showed that the plateau was not caused by the aggregation (see Supplementary Information for the results of additional experiments with suppressed aggregation).

### Sum frequency generation spectroscopy of Langmuir Monolayers

**Figure 3:**
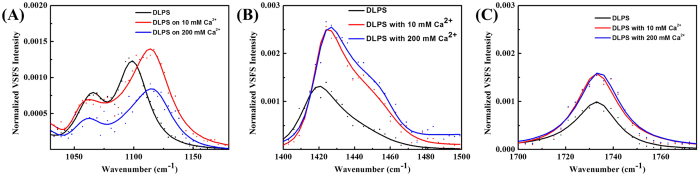
VSFS spectra of DLPS monolayers at the air/water interface at pH 7.4: Black, red and blue traces correspond to monolayers in the absence of Ca^2+^, in the presence of 10 mM Ca^2+^ and 200 mM Ca^2+^, respectively. VSFS spectrum of phosphate stretch (**A**), carboxylate stretch (**B**) and ester stretch (**C**). Solid lines are fits to the experimental spectra.

Vibrational sum frequency spectroscopy (VSFS) was used to investigate binding to carboxylate, phosphate, and carbonyl groups in DLPS monolayers. The resulting VSFS spectra of Langmuir monolayers of DLPS at the air/water interface are shown in Fig. 3. This saturated lipid was chosen for monolayer studies to avoid oxidation of double bonds over the course of the spectroscopic measurements. The measurements were made at a surface pressure of 30 mN/m with an area per lipid headgroup of 0.72 nm^2^ per lipid. The DLPS monolayers were investigated at 21 °C and were in the fluid phase under the experimental conditions which were employed. Three different spectral regions are provided, which probed (a) the phosphate symmetric stretch around 1100 cm^−1^, (b) the carboxylate stretch around 1420 cm^−1^, which also has a contribution from C-H scissoring modes, and (c) the carbonyl stretch around 1732 cm^−1^, respectively. The data provide clear evidence that calcium ions perturb the headgroup region of the monolayer. The most complex changes occur in the region of the phosphate symmetric stretch. In this case, the shoulders at 1056 cm^−1^ and 1074 cm^−1^ are from the phosphate ester stretch C-OP and from the CO-O-C, respectively^47^, while the phosphate symmetric stretch is located near 1099 cm^−1^ in the absence of Ca^2+^. The phosphate symmetric stretch peak is blue shifted by 16 cm^−1^ in the presence of Ca^2+^. Such a blue shift is evidence for dehydration and direct cation binding^48^. Significantly, the extent of this shift was essentially the same at 10 and 200 mM CaCl_2_.

The COO^−^ symmetric stretch at 1420 cm^−1^ increased in intensity in the presence of Ca^2+^. The intensity of this peak remained unchanged upon increasing the CaCl_2_ concentration to 200 mM. It should be noted that there is a shoulder on this peak near 1453 cm^−1^, which increases in intensity in the presence of Ca^2+^. These changes indicate the binding of Ca^2+^ to the carboxylate. Also, a small increase in intensity was noted in the carbonyl stretch around 1732 cm^−1^ upon introduction of 10 mM CaCl_2_ and no further significant change was noted at 200 mM CaCl_2_. This suggests that the binding of calcium ions is rather complex and that different sites are differentially occupied as the CaCl_2_ concentration increases as discussed below in the MD simulations.

### Molecular Dynamics (MD) simulations of lipid bilayers

Finally, molecular dynamics simulation of POPC and POPC/POPS bilayers at varying aqueous CaCl_2_ concentrations were used to characterize the binding at the atomic level and to explore the consequences of calcium interactions with membranes.

#### Lipid bilayers strongly adsorb calcium ions

In the course of a few tens of nanoseconds of MD simulations, all calcium ions from nominal 100 mM CaCl_2_ solution partitioned to POPC bilayer. This resulted in effectively 0 mM calcium concentration in the bulk solution, with no subsequent calcium ion desorption during 200 ns. Note that the same issue occurred in previous MD studies^18^,^26^,^31^,^33^. Here, to maintain a finite calcium concentration in the water phase, the nominal CaCl_2_ concentration was raised stepwise to 400, 600 and 700 mM. Only at the last step, was an equilibrium non-zero concentration of calcium ions in the water phase observed, with the effective 180 mM calcium concentration in water, i.e. within the concentration range explored by our experimental measurements (see Supplementary Information for more details).

Similarly, in the case of POPC/POPS bilayers, at a nominal concentration of 100 mM CaCl_2_, all calcium ions were adsorbed, whereas at a nominal concentration of 700 mM CaCl_2_ equilibrium with an effective concentration of 180 mM of calcium ions was achieved (no stepwise addition of ions was employed here). Note that since in the POPC/POPS system an additional 200 mM of calcium ions was added to neutralize PS charge, the binding capacity of this bilayer is higher than that of POPC. Under equilibrium conditions, there was 1 calcium ion adsorbed per 2 lipids in POPC/POPS and 1 ion per 3 lipids in the POPC bilayer. This is within the range of 1–4 lipids per cation as reported in earlier MD studies^18^,^26^,^33^,^49^, and 1–2 lipids per cation resulting from NMR measurements^50^. Cation exchange between membrane and water observed under these conditions demonstrates system equilibration. Time scale of this exchange strongly depends on the location of bound cation. Based on contact analysis, desorption of the ions from the lipid headgroups was observed at ~10 ns, while from lipid carbonyls only after 230–280 ns.

**Figure 4:**
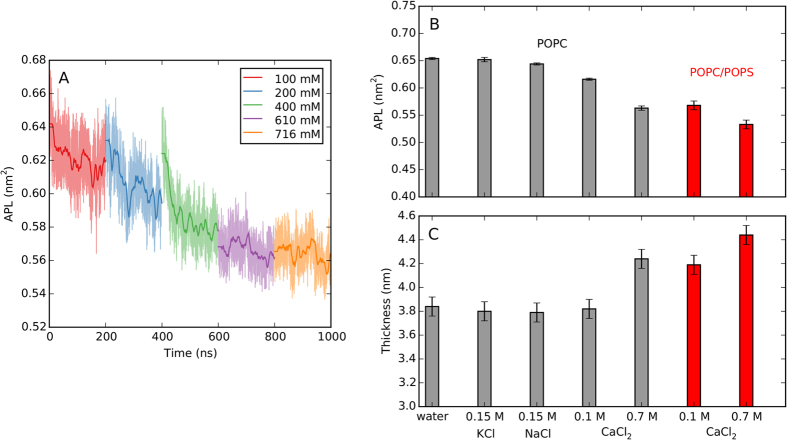
Simulated area per lipid and bilayer thickness. (**A**) Area per lipid of POPC bilayer during five MD simulations with increasing CaCl_2_ concentration. At each concentration, 200 ns-long trajectory was calculated. Final (**B**) average area per lipid and (**C**) bilayer thickness for POPC and mixed POPC/POPS bilayers in pure water and in the presence of CaCl_2_. Data for 0.15 M NaCl and KCl, taken from an additional 200 ns-long MD simulation with scaled ion charges, are given for comparison. Error bars for APL are based on block analysis. The bilayer thickness is calculated as the phosphate–phosphate distance in the density profiles with the error bars representing inaccuracy in the estimation of peak positions.

The strong binding of calcium ions resulted in lateral compression of the bilayer. The area per lipid (APL) gradually decreased after subsequent additions of CaCl_2_ (Fig. 4A). Only at nominal concentration of 700 mM CaCl_2_ was no further APL reduction observed. This corresponds to the aforementioned equilibrium between the bound and solvated calcium ions.

In the following sections, we discuss the results obtained only for two nominal CaCl_2_ concentrations: 100 mM – a low-concentration regime with 0 mM calcium ions in aqueous phase, and 700 mM – a high-concentration regime with 180 mM calcium ions in aqueous phase.

#### Calcium adsorption compresses lipid bilayers

In Fig. 4B and C, average APL and membrane thickness are reported for both POPC and POPC/POPS membranes at two CaCl_2_ concentrations and compared with effects of sodium and potassium. As mentioned above, adsorption of calcium compresses the lipid bilayer laterally, in accord with earlier MD studies^18^,^26^,^31^,^33^. In low and high CaCl_2_ concentration regimes, APL was reduced by 5 and 14% for POPC, and by 12 and 19% for POPC/POPS, respectively (Fig. 4B). Reduction of APL is accompanied by an increase of bilayer thickness (Fig. 4C). There is no direct correspondence between the two parameters, since the thickness is determined by both lateral packing of lipids and headgroup orientation. The bilayer compaction by calcium is much stronger than for monovalent cations. At 0.15 M concentration, Na^+^ and K^+^ cause almost no effect, whereas already at 0.1 M CaCl_2_ APL is altered. Membrane compression is likely caused by calcium-lipid clustering and the subsequent rearrangement of lipid molecules, just as it was observed for sodium^44^ and calcium^18^,^26^,^31^,^33^. To verify this hypothesis, we further scrutinize the changes calcium induces in the lipid bilayer at the atomic level.

#### Calcium penetrates deep affecting atomic structure of lipid bilayers

**Figure 5:**
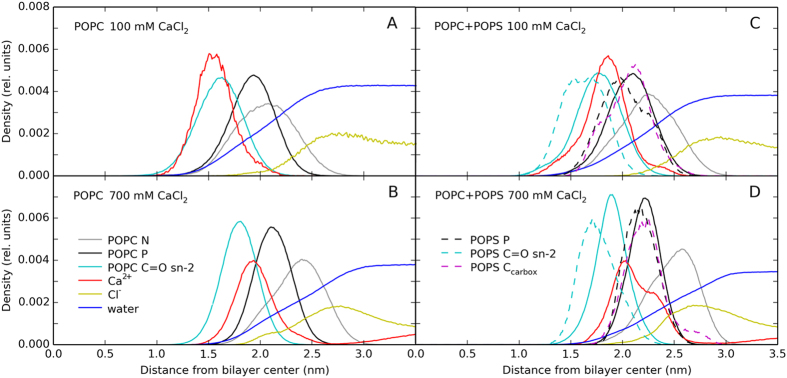
Density profiles calculated along the bilayer normal for nitrogen, phosphorous, and sn-2 carbonyl oxygen atoms of POPC and POPS, calcium and chloride ions, water, and carboxylate groups of POPS. Profiles of both bilayer leaflets are averaged.

Density profiles calculated for selected system components along the bilayer normal are depicted in Fig. 5. In the low concentration regime, calcium ions are adsorbed deeply into the POPC bilayer (Fig. 5A). Their density profile overlaps closely with that of carbonyl groups and only partially with that of the phosphate groups. To our best knowledge, significant binding to carbonyls was reported only in one earlier MD study^33^ while not observed in most other MD simulations^18^,^26^,^31^. No penetration of calcium into the purely hydrophobic membrane core was observed. Chloride ions are weakly enhanced at the water-membrane interface, likely due to their tendency to neutralize the membrane’s positive charge caused by calcium adsorption, similarly to what was previously observed in NaCl and KCl solutions^34^. A few chloride ions were able to penetrate into the headgroup region forming transient pairs with calcium ions.

In the high concentration regime, calcium ions are shifted towards the water phase. Their density peak is located half-way between those of the carbonyl and phosphate groups (Fig. 5B). Calcium ion density in the aqueous phase is clearly non-zero. The thickening of the membrane is evident from the shift of the phosphate density toward the water phase. Moreover, as a result of increased lateral packing, the carbonyl and phosphate density profiles become narrower. Separation of phosphate and choline nitrogen profiles suggests reorientation of PC headgroups. Penetration of chloride anions into the membrane headgroup region is elevated with Cl^−^ forming transient pairs with calcium ions.

In the mixed POPC/POPS bilayers, the adsorbed calcium locates between carbonyls and phosphates at both concentrations (Fig. 5C and D). With increasing CaCl_2_ concentration, the mean distance of calcium ions to the bilayer center increases only due to membrane thickening. Contrary to the POPC system, the mean distance of calcium ion to the water phase is preserved but its distribution becomes bimodal (Fig. 5D). A fraction of calcium ions locates above the mean position of the phosphate groups due to binding to COO^−^ groups of POPS. Note that the POPC/POPS membrane structure is also more complex, with POPS mostly buried below the choline groups of POPC. Contrary to previous report^23^, no lateral demixing of PC and PS was observed.

**Figure 6:**
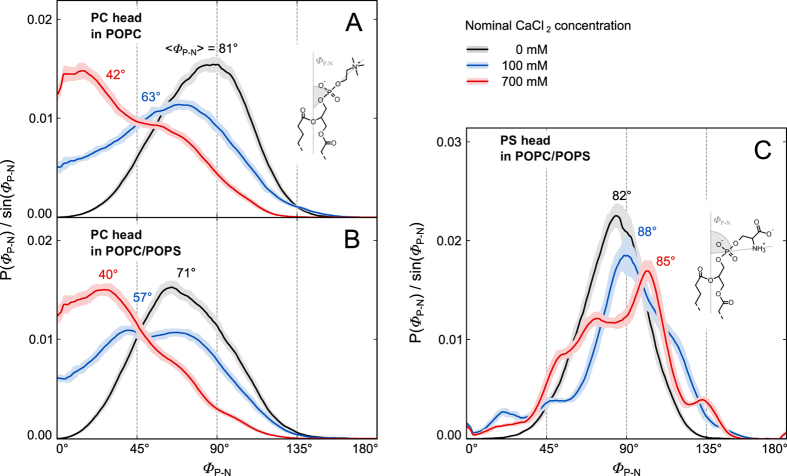
Normalized distributions of *Φ*_P–N_ headgroup angles of POPC (**A**,**B**) and POPS (**C**) in pure POPC (**A**) and POPC/POPS (**B**,**C**) bilayers for different nominal CaCl_2_ concentrations. Definitions of *Φ*_P–N_ angles and their mean values calculated based on the presented distributions, 〈*Φ*_P–N_〉, are given. The data for 0 mM CaCl_2_ are taken from ref. 44 where it was calculated for POPC in pure water, and for POPC/POPS in 1 M KCl (see text for justification). The distributions were calculated based on the final 100 ns of MD trajectories (50 ns for those from ref. 44).

Orientation of lipid headgroups was quantified by distributions of the angle between the lipid P-N dipoles and the membrane normal (Fig. 6). As suggested above, calcium ion adsorption causes a gradual rise in the POPC headgroups both in pure POPC (Fig. 6A) and in POPC/POPS bilayers, while no significant change of the mean orientation of POPS headgroups is found. Closer examination of POPS *Φ*_P-N_ distributions (Fig. 6C) and simulated trajectories reveals that while in the presence of calcium ions, PS headgroups lie on average flat with the bilayer surface, there is a fraction of PS headgroups that points both outward and inward. Hence, calcium alters the POPS headgroup orientation.

Calcium also affected hydration of the lipid bilayer. The mean number of water molecules in the vicinity of the phosphate and carbonyl groups of the lipids decreased with increasing CaCl_2_ concentration, which also corresponds to the number of adsorbed calcium ions per lipid (Table 1). Both increased lateral membrane packing and the presence of calcium ions sterically limit the number of water molecules in the headgroup and carbonyl regions of the bilayer. This result qualitatively agrees with the slight dehydration suggested in TDFS and VSFS experiments. Bilayer dehydration was also reported in earlier MD and experimental studies (e.g. refs 17, 23, 24, 25, 26).

#### Calcium-binding sites in lipid bilayers

**Figure 7:**
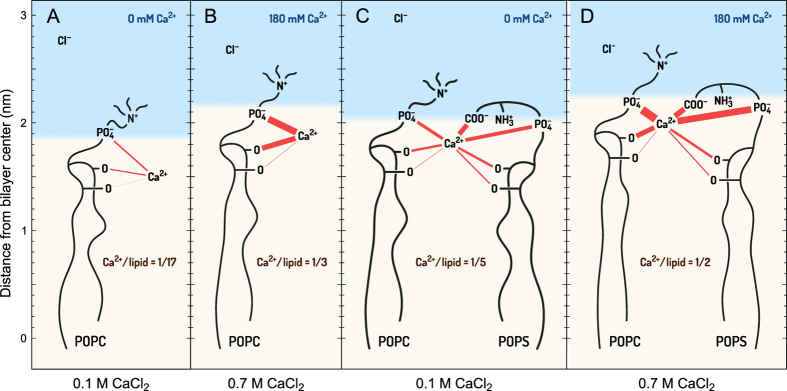
Scheme of calcium ions binding to POPC (**A**,**B**) and POPC/POPS (**C,D**) bilayers at low (**A**,**C**) and high (**B**,**D**) CaCl_2_ concentrations (accordingly, 0 and 180 mM in the water phase, corresponding to nominal 100 mM and 700 mM in the whole system). The thickness of the red lines is proportional to the average numbers of Ca^2+^ in the first coordination shell of a given group (the numbers are listed in Table S1). Positions of the functional groups, ions, and water-lipid interface (50% of bulk water density) are taken as mean positions from their density profiles (Fig. 5). Effective Ca^2+^ concentration in the water phase are given in blue. The numbers of adsorbed Ca^2+^ per total numbers of lipids are also provided.

The binding of calcium by individual atomic groups of lipids was quantified by calculating first coordination numbers (*n*_1_) from radial distribution functions – average numbers of calcium ions in the first coordination shell of a given group (Table S2). To better visualize the interactions, these numbers are schematically represented by the thickness of the red lines in Fig. 7. Ions and lipid components in Fig. 7 are placed according to their average locations calculated from Figs 5 and 6.

At low CaCl_2_ concentrations, the coordination numbers of phosphate and the *sn*-2 carbonyl groups are similar (with a slight preference for PO_4_^−^), even though calcium ions are located deep in the POPC membrane (Fig. 7A). The coordination numbers are limited by the small number of calcium ions – 1 adsorbed calcium ion per 17 POPC molecules. When the concentration increases, calcium ions shift towards the water phase (Fig. 7B). The coordination numbers increase, but their proportion preserves: 20% more calcium ions bind to PO_4_^−^ than to carbonyls. In the POPC/POPS bilayer, in the low concentration regime (Fig. 7C) calcium ions bind predominantly to the carboxylate groups of POPS (*n*_1_ = 0.58) followed by the phosphates (0.38) and carbonyls (0.34). In a number of configurations observed in simulated trajectories, a single calcium ion binds several groups of the same lipid. In POPS, calcium ions typically bind to COO^−^ and simultaneously to PO_4_^−^ and/or C=O of the same molecule. We observed that such binding caused reorientation of the POPS headgroup towards the membrane interior. High CaCl_2_ concentration modifies the equilibrium between coordination numbers, which increase to 0.68, 0.98, and 0.60 for COO^−^, PO_4_^−^ and C=O, respectively. This substantial increase in PO_4_^−^ binding is more pronounced for POPC (180% increase) than for POPS (80% increase). However, considering all contacts between calcium ions and lipids (with 0.42 nm cutoff), binding to POPS prevails. It accounts for 80 and 75% of all calcium-lipid interactions in the low and high concentration regimes, respectively.

Calcium was also concurrently bound to more than one lipid molecule at a time. Coordination numbers of the adsorbed calcium ions – mean number of the considered groups in their first coordination shell (Table 1) – reveal that at low CaCl_2_ concentration calcium clusters at least 3 lipid molecules: in POPC via PO_4_^−^ and in POPC/POPS via COO^−^. At high concentration, the clustering is somehow reduced: single calcium ion binds to 2.6 and 2.2 PO_4_^−^ in POPC POPC/POPS, respectively.

## Discussion

The principal aim of the present study was to identify calcium binding sites in zwitterionic PC and mixed PC/PS lipid bilayers and to investigate calcium-induced changes in membrane properties at the atomic level.

DLS experiments demonstrated that calcium ion adsorption at lipid membranes is guided not only by simple Coulomb interactions. Potentially, the observed clustering of lipid vesicles may be controlled by varying calcium ion concentration. TDFS measurements revealed that calcium ions restrict local mobility of various membrane regions in a concentration-dependent fashion. For example, deeply-buried carbonyl groups as well as water-exposed phosphates are subject to such restriction. These moieties can be viewed as calcium binding sites, which was further supported by VSFS experiments employing lipid monolayers where calcium ions were shown to interact with the carbonyl, phosphate, and carboxylate groups of PS lipids.

MD simulations of lipid bilayer patches were employed to get atomic level insights into calcium-membrane binding. A novel force field with scaled charges was used to account in a mean-field fashion for polarization effects and thus reduce the known ion overbinding problem of standardly used empirical force fields^35^,^51^. MD identified three calcium binding sites: carboxylate groups (POPS), phosphates, and carbonyl groups of *sn-2* lipid chains. Their relative affinities towards calcium ions vary with calcium concentration and lipid composition, but phosphates somewhat prevail over carbonyls. In PC/PS membranes, binding to carboxylate groups dominates at low calcium concentration, but is overtaken by phosphate moieties at higher concentration. Calcium binding leads to lipid clustering, lateral shrinkage and thickening of the bilayer, which is more pronounced for negatively charged PC/PS membranes. This is in full agreement with the restricted carbonyl mobility probed by TDFS. As has been shown, these parameters are often strongly correlated^52^. MD shows that lateral compression leads to membrane dehydration, which was also suggested in VSFS and TDFS experiments. Our findings are in overall agreement with previous experimental and computational studies of calcium-lipid bilayer systems reporting on strong propensity of calcium toward negatively charged bilayers accompanied by significant membrane remodeling, as discussed in the Introduction. By combining several experimental techniques with MD simulations that employ the novel force field, we demonstrate and evaluate how propensity of calcium binding sites changes with ion concentration. Of note, in contrary to most of previous MD studies, we show by both simulations and experiments that carbonyl groups of lipids are important calcium binders, in particular at low concentrations.

Our results demonstrate that calcium-bilayer interactions are complex and specific. We postulate that in a biological context, some of the phenomena accompanying calcium ion binding by lipid membranes may play a considerable role. First, a high affinity of calcium toward membranes is important from the point of view of calcium signaling. It allows the negatively charged inner leaflet of cellular membrane to act as a calcium buffer and modulate calcium diffusivity in calcium signaling microdomains. Second, the variety of calcium binding sites and their energetic heterogeneity can play a role in synaptic plasticity related to so-called residual calcium^53^. Third, calcium ion-induced changes in lipid dynamics and structure can play an important biological role, i.e. reduced lipid mobility can influence membrane receptors and headgroup rearrangement can change the affinity of phospholipid-binding proteins. Fourth, lipids on the millisecond timescale, characteristic for calcium signaling, are laterally mobile and can serve as mobile calcium buffers by transporting the cations out of the proximity of a calcium channel. Last but not least, the ability of calcium ions to overcharge PC/PS bilayers may potentially play a significant role for vesicle trafficking, membrane fusion, and membrane-protein binding by modulation of the effective charge of the inner leaflet of cellular membrane.

## Conclusions

Calcium ion binding sites are heterogeneous both from the point of view of binding affinity and their positioning in the membrane. The character of the calcium binding varies with calcium concentration; this issue is of particular importance as significant concentration spikes of calcium ions occur along calcium signaling pathways. The present results support the conjecture that lipid membranes, in particular the negatively charged inner leaflet of the plasma membrane, can act as effective calcium buffers upon calcium ions entering the cytosol. Of equal importance is the fact that the strong binding of this ion significantly alters the membranes by means of reduction of their hydration, lipid mobility, and lateral inter-lipid distance. Such local conformational membrane remodeling may play a significant role in modulation of lipid-protein interactions as well as membrane-membrane interactions. Overall, the phenomena related to calcium ions-membrane interactions demonstrated here point to their diverse roles in biological systems.

## Experimental

### Materials

1,2-dioleoyl-*sn*-glycero-3-phosphocholine (DOPC), 1,2-dioleoyl-*sn*-glycero-3-phospho-L-serine (DOPS), 1,2-dilauroyl-*sn*-glycero-3-phospho-L-serine (DLPS), and 1,2-dioleoyl-*sn*-glycero-3-phosphoethanolamine-N-[methoxy(polyethylene glycol)-2000] (DOPE-PEG2000) were supplied by Avanti Polar Lipids, Inc. (Alabaster, USA). 6-lauroyl-2-dimethylaminonaphthalene (Laurdan) was obtained from Molecular Probes (Eugene, USA). 4-[(n-dodecylthio)methyl]-7-(N,N-dimethylamino)-coumarin (Dtmac) was a gift of prof. Kraayenhof, Vrije Universiteit Amsterdam. CaCl_2_ with purity ≥99% was used. All chemicals were used without further purification.

### Liposome preparation

Appropriate volumes of lipids and fluorescent probe stock solutions were mixed in glass tubes, dried under a nitrogen stream and left for at least 2 hours in vacuum. Lipid films were rehydrated in 1.5 mL MiliQ water (Milipore, USA) containing either 0.1 mM ethylenediaminetetraacetic acid or appropriate concentration of CaCl_2_. Liposomes in the form of large unilamellar vesicles were formed by extrusion through a 100 nm pore diameter membrane filters (Avestin, Ottawa, Canada). The final lipid concentration was 1 mM with 1:100 (mol/mol) fluorescent probe to lipid ratio.

### DLS and zeta potential

The size and zeta potential of the liposomes were measured by DLS. The samples were transferred to UV grade poly(methyl methacrylate) cuvettes, or to the so-called “dip” cell (Malvern Instruments Ltd., Worcestershire, UK) for zeta-potential measurements, and equilibrated at 298 K for 3 minutes before each measurement. The light scattering setup of Zetasizer Nano ZS (Malvern Instruments Ltd.) consisted of a He–Ne laser (532 nm) and an avalanche photodiode detector (APD). The scattering intensity was collected at an angle of 173°. The nano-ZS automatically adapts to the sample by adjusting the intensity of the laser and the attenuator of the photomultiplier. Intensity-weighted size distributions were obtained using regularized fitting implemented in Zetasizer Software 6.2 (Malvern Instruments Ltd.).

### TDFS method

Samples in 1.5 mL quartz cuvettes were equilibrated for 15 minutes and measured at 283 K. The temperature was stabilized using a water circulating bath. Steady-state emission spectra were recorded on a Fluorolog-3 spectrofluorometer (model FL3–11; JobinYvon Inc., Edison, NJ, USA) equipped with a xenon-arc lamp. Time-resolved measurements were performed on a 5000U Single Photon Counting setup using a cooled Hamamatsu R3809U-50 microchannel plate photomultiplier (IBH, UK). An emission cutoff filter (>399 nm) was used to eliminate the scattered light. Fluorescent probes were excited at 373 nm with the IBH NanoLed 11 laser diode and fluorescent decays were collected at 400 to 550 nm with 10 nm steps. Each decay was fitted with a multi-exponential function using the iterative reconvolution procedure (IBH DAS6 software). The decays and the steady-state emission spectrum were used to reconstruct time-resolved emission spectra, which were then fitted with log-normal function in order to determine position of their maxima, ν(t), and their width^54^.

The TDFS method is based on monitoring the Stokes shift kinetics of polarity sensitive probes. The dipolar relaxation of the microenvironment of the probe is mapped in the recorded time-resolved emission spectra, which carry information about the microenvironment polarity and mobility. These two properties can be quantified by total spectral shift (Δ*ν*), and solvent relaxation time (*τ*), respectively. It has been shown in lipid bilayers that these two parameters are related to the hydration and local mobility of the hydrated lipid moieties^55^,^56^. Δ*ν* is calculated as Δ*ν* = *ν*(0) − *ν*(∞), where *ν*(0) is spectrum position immediately after electronic excitation, and *ν*(∞) is spectrum position after dipolar relaxation of solvent is completed. *ν*(0) was estimated according to ref. 57 to be 23800 cm^−1^ and 22750 cm^−1^, respectively for Laurdan and Dtmac. The spectral shift is proportional to the loss of energy due to dipole reorientation, therefore, corresponds to the polarity of the probe environment. The second parameter, the so-called integrated relaxation time, 
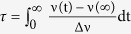
, is used to characterize the kinetics of the relaxation. It was shown to reflect mobility of the hydrated lipid moieties in the vicinity of the probe. Intrinsic uncertainty for Δν was 50 cm^−1^ and 0.05 ns for *τ*.

### VSFS spectroscopy

In vibrational sum frequency generation, a fixed wavelength visible beam (532 nm) and a tunable IR beam were spatially and temporally overlapped at a phospholipid/water interface to generate the sum frequency beam. The intensity of the sum frequency radiation is proportional to the square of second order nonlinear susceptibility and the intensity of the visible and IR beams: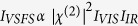, where *χ*^(2)^ is the second order nonlinear susceptibility, *I*_*VIS*_*, I*_*IR*_ and *I*_*VSFS*_ are the intensities of the visible, infrared and sum frequency beams respectively^58^. The VSFS experimental setup (purchased from EKSPLA, Lithuania) consisted of a 1064 nm Nd:YAG laser (pulse duration: 30 ps; pulse energy: 40 mJ; maximum repetition rate 50 Hz), which was directed to the harmonic unit (H500). The second harmonic (532 nm) and fundamental beams from the harmonic unit pumped the optical parametric generator/difference frequency generator (PG501/DFG) unit. The infrared beam was tuned between 1000 and 4000 cm^−1^ and the spectral resolution was <6 cm^−1^. VSFS experiments were performed in a Langmuir trough (NIMA technology, England) made from Teflon and equipped with a pressure sensor and Teflon barriers. The trough had a total area of 65 cm^2^ and a subphase volume of 35 mL. Langmuir monolayers of lipid molecules were first formed at the air/water interface and the VSFS spectra were then collected in the absence and presence of metal ions in the subphase. The IR and visible beams had incident angles of 55° and 60°, respectively. These were spatially and temporally overlapped at air/water interface and the sum frequency signal was collected at an angle of approximately 59°.

Vibrational sum frequency spectra of Langmuir monolayers of DLPS were monitored in the presence of calcium ions using the ssp polarization combination (referring to the sum frequency, visible, and infrared polarizations, respectively). The surface pressure and temperature were maintained at 30 mN/m and 21 °C, respectively, throughout all the spectroscopic experiments. The subphase was buffered using 10 mM Tris at pH of 7.4. The VSFS signal was collected at 3 cm^−1^ intervals and each point on the spectrum represented an average of 600 laser pulses. The intensity of the collected VSFS signal was normalized to the corresponding IR and visible intensities. Each spectrum represents an average of five measurements.

### MD simulations

MD simulations were performed for pure 1-palmitoyl-2-oleoyl-*sn*-glycero-3-phosphocholine (POPC) and its mixture with 1-palmitoyl-2-oleoyl-*sn*-glycero-3-phospho-L-serine (POPS): POPC/POPS 4:1, mol:mol. Several nominal calcium concentrations with respect to the number of water molecules in the simulation box, were considered. Table S3 in the Supplementary Information summarizes the composition of all simulated systems. Each lipid bilayer consisted of 128 lipid molecules (64 in each leaflet) hydrated with over 4300 water molecules. The mixed POPC/POPS system was prepared by replacing 12 randomly chosen PC headgroups in each leaflet of pre-equilibrated POPC bilayer with PS. To neutralize the system, 16 calcium ions were added to the water phase (each with scaled charge of +1.5, see the charge scaling details below). Additional calcium and chloride ions were added to obtain the required salt concentration (not counting the neutralizing cations). The presence of the additional neutralizing cations resulted in nominal 306 mM and 919 mM calcium concentrations in POPC/POPS system. 10 ns-long pre-equilibration MD runs were performed, followed by 200 ns and 300 ns-long trajectories for POPC and POPC/POPS systems, respectively. Only the last 100 ns of each trajectory were used for time independent analysis.

A standard united-atom non-polarizable Berger’s force field was employed for the description of the lipids^59^. The simple point charge (SPC) water model used is compatible with the Berger’s force field^60^. A recently developed set of parameters was used for calcium and chloride ions; the parameters derived by employing charge scaling were shown to improve ionic interactions in aqueous environments^35^. The basic idea of this approach is to include electronic polarization in a mean-field manner via rescaling the ionic charges by the inverse of the square root of the electronic part of the water dielectric constant, i.e by a factor of 0.75^61^,^62^. Hence, the resulting charges of calcium and chloride ions in simulation are +1.5e and −0.75e, respectively. As a mean-field ansatz, such an approach is applicable to homogeneous media in terms of the electronic polarization. Note that none of currently available combinations of ion and lipid force fields are able to fully quantitatively account for calcium-phospholipids binding. Nevertheless, the scaled charges employed here seem to, at least, semi-quantitatively describe these interaction. Further details of the MD methodology, force fields tests and issues, and discussion of the ion charge scaling is given in the Supplementary Information.

## Additional Information

**How to cite this article**: Melcrova, A. *et al*. The complex nature of calcium cation interactions with phospholipid bilayers. *Sci. Rep.* **6**, 38035; doi: 10.1038/srep38035 (2016).

**Publisher's note:** Springer Nature remains neutral with regard to jurisdictional claims in published maps and institutional affiliations.

## Supplementary Material

Supplementary Information

## Figures and Tables

**Table 1:** Adsorption of Ca^2+^ (extent and coordination numbers) and lipid hydration.

Lipid bilayer	[CaCl_2_]	Lipids per Ca^2+^_abs._(a)	Ca^2+^ coordination numbers(b)			Hydration(c)	
			PO_4_	C=O	COO^−^	PO_4_	C=O
POPC	0.1 M	17 ± 1	3.0 ± 0.5	2.5 ± 0.5	—	3.5 ± 0.1	2.0 ± 0.1
	0.7 M	3 ± 1	2.6 ± 0.5	2.1 ± 0.5	—	2.4 ± 0.1	1.3 ± 0.1
POPC/POPS (4:1, mol:mol)	0.1 M	5 ± 1	2.0 ± 0.5	1.8 ± 0.5	3.0 ± 0.5	3.0 ± 0.1	1.7 ± 0.1
	0.7 M	2 ± 1	2.2 ± 0.5	1.3 ± 0.5	1.5 ± 0.5	2.2 ± 0.1	1.4 ± 0.1

^(a)^Ratio of the total number of lipids in the system per number of adsorbed calcium cations.

^(b)^Average number of the considered groups in the first coordination shell of an adsorbed Ca^2+^.

^(c)^Number of water molecules per lipid in the first solvation shell of selected atom groups (cutoff determined from radial distribution functions as 2.4 Å for carbonyl oxygens and 3.35 Å for phosphate phosphors); the given uncertainties represent SD.
