# Supplementary material for: The complex nature of calcium cation interactions with phospholipid bilayers
Source: Sci Rep. 2016 Dec 1;6:38035. doi: 10.1038/srep38035 (PMC5131315; doi:10.1038/srep38035)
Supplement: Supplementary Information [file srep38035-s1.pdf]

Supplementary information

The complex nature of calcium cation interactions with phospholipid bilayers

Adela Melcrova[1], Sarka Pokorna[1], Saranya Pullanchery[2], Miriam Kohagen[3,4], Piotr Jurkiewicz[1,*], Martin Hof[1], Pavel Jungwirth[3,5], Paul S. Cremer[2,6,*], and Lukasz Cwiklik,[1,3,*]

[1]J. Heyrovský Institute of Physical Chemistry, Academy of Sciences of the Czech Republic, v.v.i., Dolejškova 3, Prague, 18223, Czech Republic
[2]Department of Chemistry, Pennsylvania State University, University Park, PA 16802, United States
[3]Institute of Organic Chemistry and Biochemistry, Academy of Sciences of the Czech Republic, Flemingovo nám. 2, Prague, 16610, Czech Republic
[4]Institute for Computational Physics, University of Stuttgart, Allmandring 3, Stuttgart, 70569, Germany
[5]Department of Physics, Tampere University of Technology, POB 692, Tampere, FI-33101, Finland
[6]Department of Biochemistry and Molecular Biology, Pennsylvania State University, University Park, PA 16802, United States
[*]*corresponding authors: psc11@psu.edu (P.C.), piotr.jurkiewicz@jh-inst.cas.cz (P.Jur.), and lukasz.cwiklik@jh-inst.cas.cz (L.C.)*

Table of Contents

1. TDFS – suppression of vesicle aggregation

2. TDFS – POPC/POPS vs DOPC/DOPS

3. VSFS – spectral fitting

4. MD – Calcium cations adsorption vs time

5. MD – Coordination numbers

6. MD – Composition of the Simulated Systems

7. MD – Charge Scaling

8. MD – Charge Scaling and Water Models

9. MD – Detailed Simulation Setup

10. References

1. TDFS – suppression of vesicle aggregation

It is striking that the plateau of τ observed for Laurdan in DOPC/DOPS at 5-50 mM $CaCl_2$ coincide with the aggregation of those vesicles at 5-30 mM $CaCl_2$ reported by DLS. To test whether these phenomena are causally related, we coated the vesicles with polyethylene glycol (PEG), which sterically prevents their aggregation.[1] Indeed, the vesicles composed of DOPC/DOPS/DOPE-PEG (75/20/5, mol/mol/mol) were monodisperse over the whole $CaCl_2$ concentration range. The TDFS data for DOPC/DOPS/DOPE-PEG are included in Fig. S1 (dashed curves). While all the τ values are elevated, the trends measured for the DOPC/DOPS system persist for DOPC/DOPS/DOPE-PEG as well. Preservation of the plateau of τ for Laurdan verifies that it is not a result of vesicle aggregation. The slopes of τ become only slightly steeper; ~65 and ~0.9 ns/M, for the 1-5 mM and 50-1000 mM $CaCl_2$ ranges, respectively (compared to ~55 and ~0.5 ns/M in vesicles with no PEG present). This can be attributed to the slightly increased negative surface charge of the vesicles due to the addition of 5 mol% of negatively charged DOPE-PEG, which is likely to attract more calcium cations. PEGylation in the absence of calcium also restricts local lipid mobility, which is more pronounced at the phosphate groups, which are located closer to the PEG chains. Relaxation probed by Dtmac slows down ~40 % irrespective of the $CaCl_2$ concentration. For Laurdan this slowdown increased from 7 % in the absence of calcium to 20 % at 1 M $CaCl_2$. This is in agreement with the hindered lateral lipid diffusion in PEGylated lipid membranes.[2] While the changes in $\Delta\nu$ are very small, it seems that PEGylation slightly dehydrates the carbonyl region.

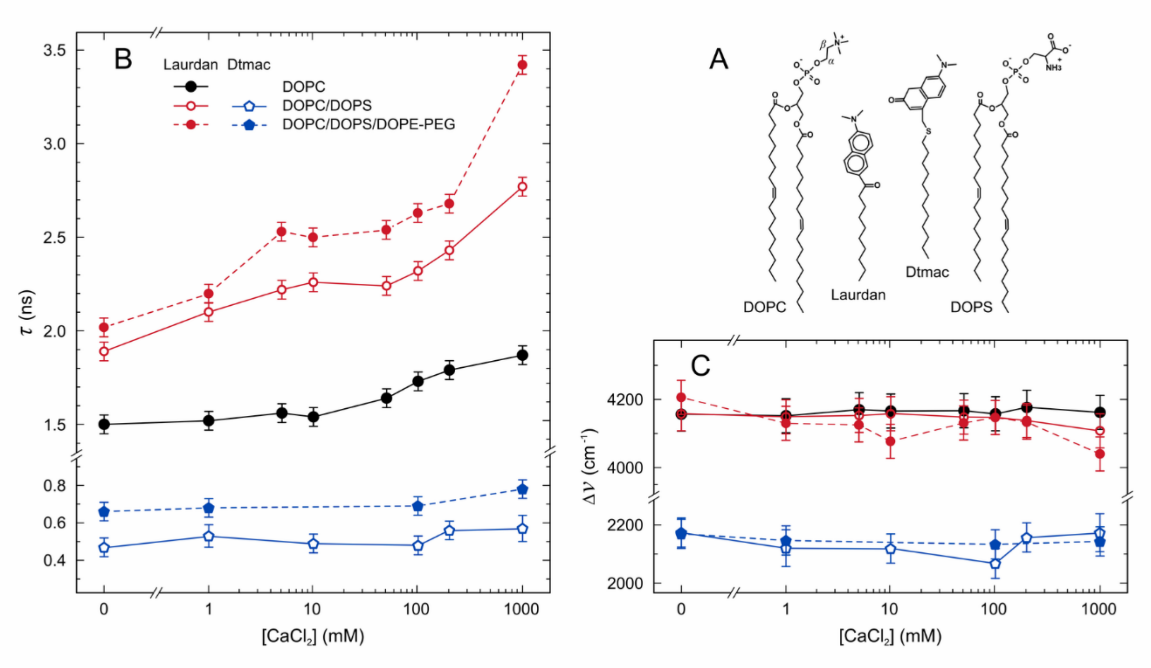

Figure S1. Laurdan and Dtmac TDFS measured at DOPC, DOPC/DOPS and DOPC/DOPS/DOPE-PEG LUVs. (A) Location of the probes with respect to DOPC and DOPS molecules in membranes. (B) Integrated relaxation time, τ, and (C) total spectral shift, Δν. 1 mol% of the probes were embedded in LUVs of various composition dispersed in water or in $CaCl_2$ solutions of various concentrations (1-1000 mM). Measured at 283 K; error bars represent SD, n ≥ 2.

2. TDFS – POPC/POPS vs DOPC/DOPS

The lipids used in TDFS experiments (DOPC, DOPS) presented herein have double unsaturated oleoyl chains instead of mixed oleoyl and saturated palmitoyl chains of POPC and POPS used in MD simulations. The reason for this choice is as follows. The presence of saturated chains makes POPC/POPS bilayer more rigid, and the relaxation time of Laurdan becomes considerably longer than in DOPC/DOPS bilayer: 3.17 ns and 1.89 ns, for the two systems respectively; both with 20 mol% of PS, measured at 10 °C in the absence of calcium. Addition of $CaCl_2$ further rigidifies the glycerol level of POPC/POPS bilayers resulting in very slow relaxation of Laurdan that can no longer be as precisely described as in DOPC/DOPS system. For the specific calcium effects the aliphatic chains of phospholipid should not be the main determinants as long as the headgroup is identical and the lipid tails are not vastly different. Nevertheless, POPC/POPS system was also measured for a comparison. Fluorescence dynamic spectral shifts for both lipid systems are shown in Fig. S2. The TDFS results obtained for POPC/POPS agree well with those of DOPC/DOPS system, and the changes between the different calcium concentrations are more pronounced (although the obtained results have larger uncertainties due to the above mentioned problems with data analysis).

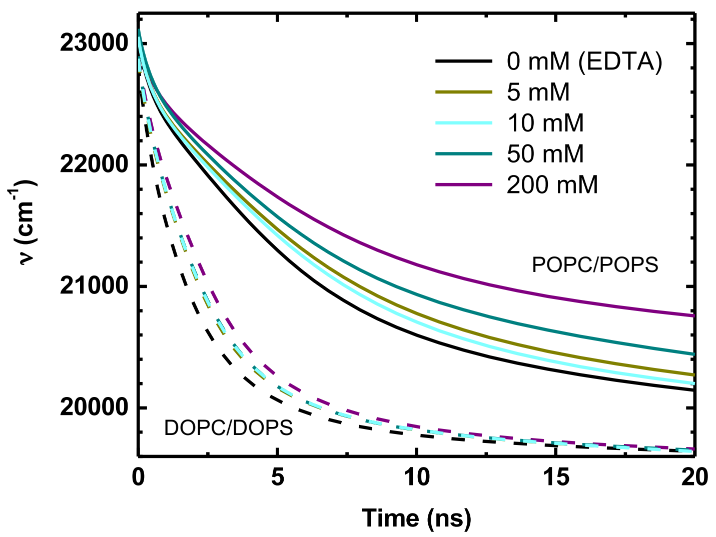

Figure S2. Comparison of Laurdan TDFS results obtained for POPC/POPS and DOPC/DOPS systems in the presence of different $CaCl_2$ concentrations. The presented relaxation curves represent the position of the Time-Resolved Emission Spectra of Laurdan given in reciprocal centimeters.

3. VSFS spectral fitting

The intensity of the sum frequency signal follows equation 1,

$$I_{SFG} \propto |\chi_{eff}^{(2)}| \times I_{Vis} \times I_{IR} \quad (1)$$

where I_{Vis} and I_{IR} are the intensities of the incoming visible and infrared laser beams, respectively. $|\chi_{eff}^{(2)}|$ represents the second order nonlinear susceptibility, which can be further expressed as:

$$\chi_{eff}^{(2)} = \chi_{NR}^{(2)} + \chi_{R}^{(2)} = \chi_{NR}^{(2)} + \sum_q \frac{A_q}{\omega_{IR} - \omega_q + i\Gamma_q} \quad (2)$$

where $\chi_{NR}^{(2)}$ and $\chi_{R}^{(2)}$ are the frequency independent non-resonant susceptibility term and the frequency dependent resonant susceptibility term, respectively. $\chi_{R}^{(2)}$ of the qth resonant mode is a function of the oscillator strength, A_q, resonant frequency, ω_q, peak width, Γ_q, and the frequency of the input infrared laser beam, ω_{IR}. The spectra have each been normalized to the intensities of incoming visible and IR beams. The normalized spectra were then fit to equation 2 by a computer program using MATLAB software. The fitted oscillator strengths, A_q, and the peak widths, Γ_q for DLPS are listed in Table S1.

Table S1. Fit parameters for the DLPS spectra

Sample		DLPS on 10 mM Tris	DLPS on 10 mM Ca^{2+}	DLPS on 200 mM Ca^{2+}
C-OP stretch	ω_q (cm^{-1})<br>A_q<br>Γ_q (cm^{-1})	1050 ± 2<br>-2.99 ± 0.4<br>34 ± 1	1049 ± 4<br>-0.95 ± 1<br>16 ± 1	1048 ± 1<br>-1.8 ± 0.05<br>26 ± 2
CO-O-C ss	ω_q (cm^{-1})<br>A_q<br>Γ_q (cm^{-1})	1070 ± 0.5<br>0.53 ± 0.1<br>16 ± 2	1069 ± 0.5<br>0.02 ± 0.04<br>13 ± 1	1066 ± 4<br>0.2 ± 0.2<br>16 ± 4
PO_2-ss	ω_q (cm^{-1})<br>A_q<br>Γ_q (cm^{-1})	1099<br>0.74 ± 0.04<br>18 ±1	1116 ±2<br>0.72 ± 0.1<br>20 ± 2	1119 ± 1<br>0.6 ± 0.1<br>20 ± 2
COO^- stretch	ω_q (cm^{-1})<br>A_q<br>Γ_q (cm^{-1})	1417 ± 4<br>0.5 ± 0.06<br>13 ± 0.5	1421± 1<br>0.5 ± 0.07<br>11 ± 0.6	1422 ± 1<br>0.5 ± 0.1<br>12 ± 1
COO^- stretch or C-H bending	ω_q (cm^{-1})<br>A_q<br>Γ_q (cm^{-1})	1452 ± 4<br>-0.4 ± 0.1<br>27 ± 5	1459 ± 3<br>-0.43 ±0.3<br>21 ± 1	1460 ± 4<br>-0.24 ±0.4<br>15 ± 10
C=O stretch	ω_q (cm^{-1})<br>A_q<br>Γ_q (cm^{-1})	1733<br>0.3 ± 0.04<br>11	1733 ± 1<br>0.43 ± 0.02<br>11	1734 ± 0.5<br>0.43 ± 0.05<br>11

4. MD – Calcium cations adsorption vs time

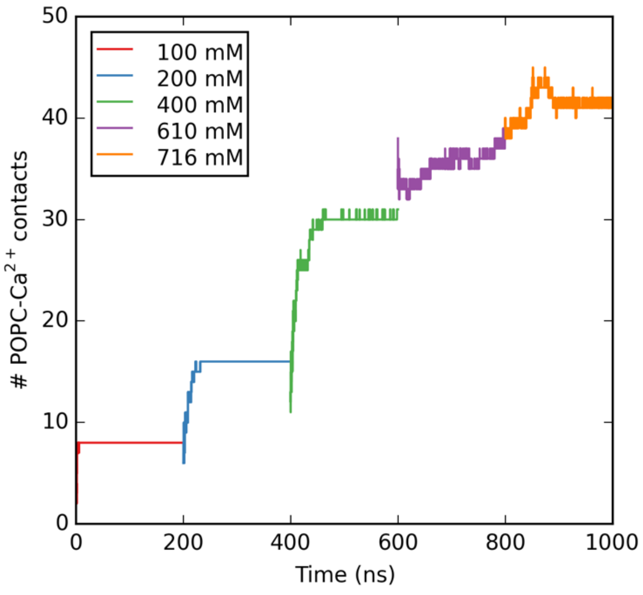

Figure S3. Number of lipid-Ca^{2+} contacts (with the 0.42 nm cutoff) calculated along simulated trajectories with POPC bilayer.

At 100, 200 mM calcium concentration, all cations were irreversibly adsorbed at the membrane on the 200 ns simulation timescale (see Fig. S3). At 400 mM, there was one ion not reversibly adsorbed. At 610 mM, most of cations were adsorbed irreversibly within 200 ns with some still remaining in the water phase. A prolonged trajectory (>200 ns) demonstrated that the remaining ions eventually adsorbed irreversibly. Only at 716 mM, absorption/desorption equilibrium was achieved with significant and stable ion population present in the water phase, as demonstrated by ion-lipid contacts. At 716 mM, the average number of cations residing in the water phase under equilibrium was accounting for the concentration of 180 mM.

5. MD – Coordination numbers

Table S2. Coordination numbers of lipid membrane groups by Ca^{2+} (average number of Ca^{2+} in the first coordination shell of the group). Only adsorbed ions are taken into account. The data for both POPC and POPC/POPS systems were calculated at two $CaCl_2$ concentrations upon system equilibration. The cutoff of 0.42 nm was employed for calcium-phosphorous atom contacts while the calcium-oxygen contacts with both carbonyl and carboxylate groups were cut off at 0.3 nm. These cut off values were chosen to match the localization of the first minimum of the corresponding radial distribution functions. Standard error values are reported.

	POPC 100 mM Ca^{2+}	POPC 716 mM Ca^{2+}	POPC/POPS 102 mM Ca^{2+}	POPC/POPS 715 Ca^{2+}
POPC PO_4	0.18 ± 0.01	0.83 ± 0.01	0.37 ± 0.01	1.01 ± 0.02
POPC *sn-2* C=O	0.15 ± 0.01	0.65 ± 0.02	0.28 ± 0.01	0.60 ± 0.02
POPC *sn-1* C=O	0.001 ± 0.003	0.030 ± 0.007	0.040 ± 0.007	0.04 ± 0.02
POPS PO_4	-	-	0.45 ± 0.02	0.82 ± 0.05
POPS *sn-2* C=O	-	-	0.28 ± 0.03	0.29 ± 0.04
POPS *sn-1* C=O	-	-	0.15 ± 0.03	0.13 ± 0.03
POPS COO^-	-	-	0.58 ± 0.05	0.68 ± 0.05
all PO_4	0.18 ± 0.01	0.83 ± 0.01	0.38 ± 0.01	0.98 ± 0.02
all C=P	0.15 ± 0.01	0.68 ± 0.02	0.34 ± 0.02	0.60 ± 0.03

6. MD – Composition of the Simulated Systems

Table S3. Composition of simulated systems. The two first rows show the concentrations of $CaCl_2$ and scaled calcium ions with respect to the number of water molecules; the next rows contain number of molecules and ions present at each system.

	POPC	POPC	POPC/POPS	POPC/POPS
conc. $CaCl_2$ [mM]	100 mM	716 mM	102 mM	715 mM
conc. $Ca^{1.5+}$ [mM]	100 mM	716 mM	306 mM	919 mM
POPC	128	128	104	104
POPS	--	--	24	24
water	4452	4308	4306	4306
neutralizing $Ca^{1.5+}$	--	--	16	16
additional $Ca^{1.5+}$	8	56	8	56
sum of $Ca^{1.5+}$	8	56	24	72

7. MD – Charge Scaling

Lipid molecules were described using the united-atom Berger force field.[3] For Ca^{2+} and Cl^- ions, three force fields were tested: non-scaled standard GROMOS, scaled force field without tuned van-der-Waals radii (ECC), and the scaled one with vdW parameters adjusted (ECCR) according to neutron scattering data from literature.[4] The ECCR was recently developed by Kohagen et al. and was shown to perform better than other parameter sets in concentrated water solutions of ions.[5] This is especially true for the structure but also other properties like the self-diffusion coefficients were well reproduced in comparison to experiment. A recent study shows, that protein-ion interactions are better described using scaled charges, too.[6] The SCP water model was employed (see also the discussion of the water force field issues below).[7] The force field tests were performed in the system containing a POPC bilayer hydrated with 100 mM $CaCl_2$ solution.

As demonstrated in earlier works on cation-membrane interactions, a relatively long simulation time is required for system equilibration.[8,9] Indeed, for each force field employed here, at least 100 ns were needed for equilibration (see Fig. S4). Therefore, last 100 ns of the 200 ns-long trajectories were used in time-averaged analysis of MD simulations. For all considered force fields, the area per lipid and membrane thickness, the latter defined as the phosphate–phosphate distance in the density profile, are the same within the error range. Namely, area per lipid is equal to 0.61 (± 0.2) nm^2, while membrane thickness is equal to 3.9

(± 0.1) nm. Hence, no difference between the force fields is noticeable in terms of these two global membrane properties. Similarly, there are no significant differences in density profiles calculated in these three systems (see Fig. S5).

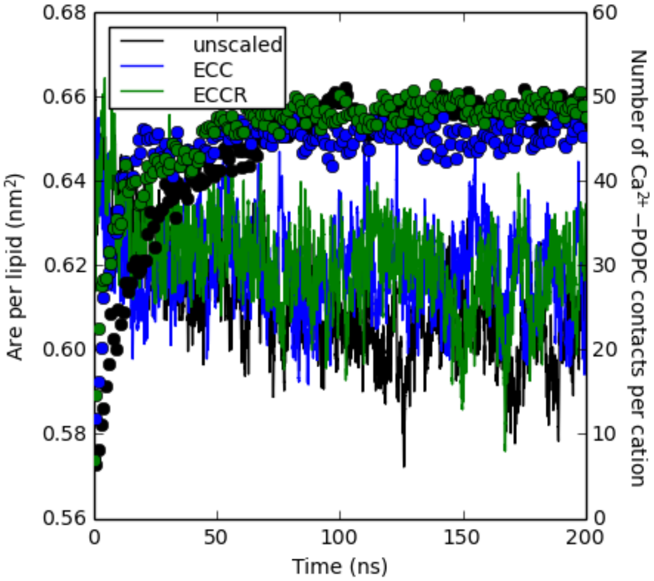

Fig. S4. Area per lipid (lines) and number of Ca^{2+}–lipid contacts (circles) as a function of MD simulation time. The cutoff of 0.6 nm was employed for cation-POPC contacts calculation.

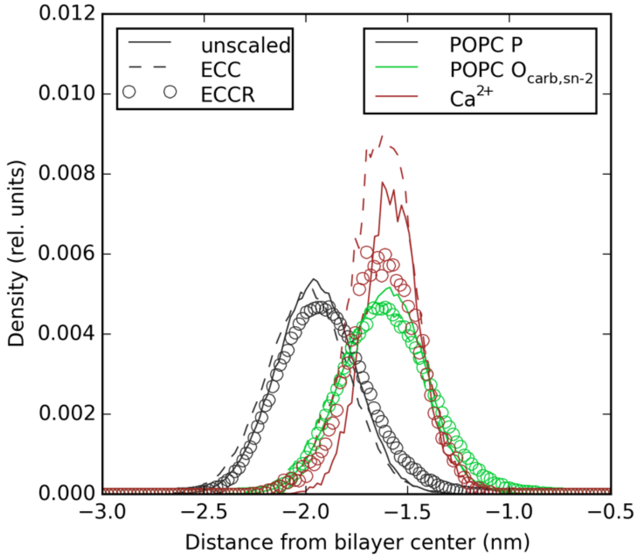

Fig. S5. Density profiles of phosphate, sn-2 carbonyl oxygen, and calcium cation calculated in the three considered force fields. For clarity, the data for one leaflet are presented. The density profiles are approximately symmetric with respect to the bilayer midplane.

Qualitative differences between the force fields occur regarding calcium binding sites. While in each case calcium significantly binds carbonyl oxygen atoms of sn-2 acyl chains of POPC (as demonstrated by radial distribution functions in Fig. S6), the extent of this binding depends on the force field. Namely, in the 100 mM $CaCl_2$ employed here, the number of carbonyl oxygen atoms present in the first coordination shell of an average calcium cation is equal to 4.1 for the unscaled force field; it is reduced to 3.2 in the case of ECC; and diminishes to 2.5 in ECCR (it is assumed here that the first coordination shell comprises of the atoms located in the distance up to the position of the first minimum at the corresponding radial distribution function). The most significant difference between the force fields arises regarding the binding of calcium to phosphate groups. Specifically, while calcium binds phosphate groups negligibly in

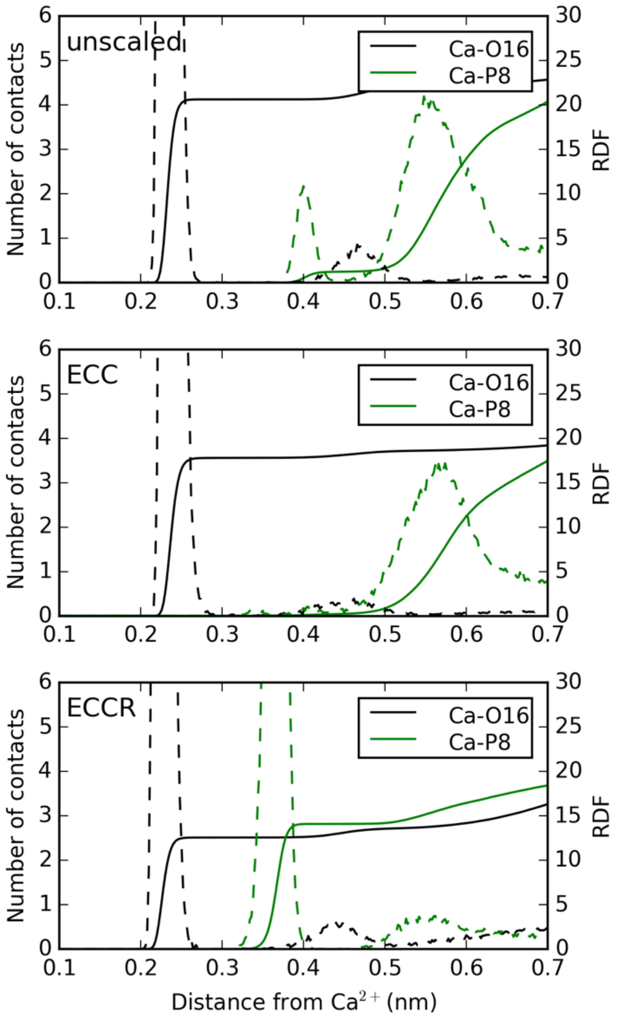

Fig. S6. Number of contacts (solid lines) and radial distribution function (dashed line) of Ca^{2+} with sn-2 carbonyl and phosphate groups of POPC for different force field parameters of calcium cation (100 mM CaCl2 with POPC).

the case of both unscaled and ECC parametrizations, a strong binding occurs in the case of ECCR where, on average, Ca^{2+} binds 2.9 phosphate groups in the first coordination shell. Note that the latter is somewhat higher than the number of bound carbonyl groups employing ECCR. To further elucidate on the calcium-membrane binding in the considered force fields, we calculated a free-energy profile of Ca^{2+} interaction with the POPC membrane (see Fig. S7). The free-energy in the case of all considered models is the same within the error (~5±2 kcal/mol). Importantly, the shape of the free-energy well is broader in the ECCR in comparison with the two remaining force fields; this is due to a significant binding of Ca^{2+} to the phosphate groups which are located at the very membrane-water interface.

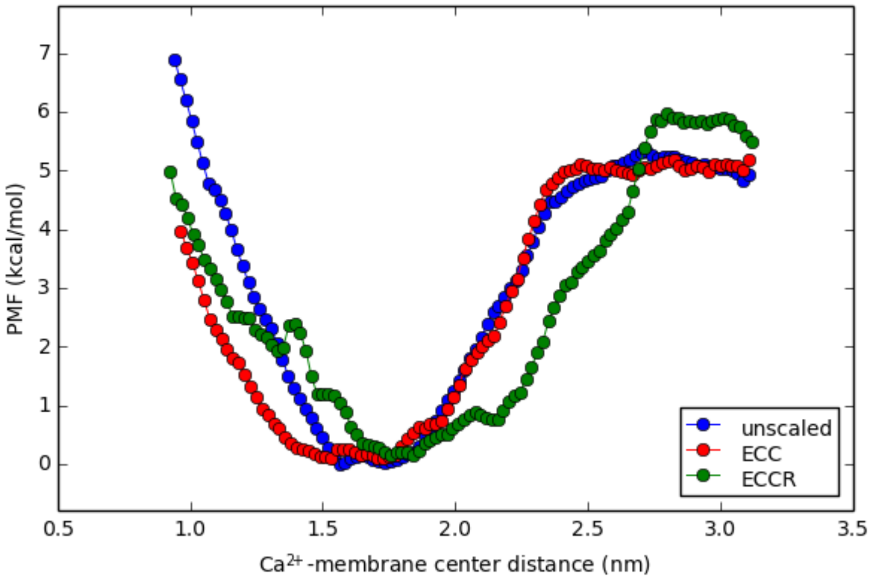

Fig. S7. Free-energy (potential of mean force – PMF) as a function of the distance between the calcium cation and the center of the POPC lipid bilayer for various force fields. Calculations were performed employing the umbrella sampling method for membranes hydrated with the 150 mM KCl solution in the presence of one Ca^{2+} cation. Umbrella windows were spaced each 0.1 nm with the calcium-bilayer distance restrained employing the harmonic force (with force constant of 3000 kJ mol^{-1} nm^{-2}). A trajectory of 10 ns was calculated in each window with the final 4 ns used for the potential of mean force calculation. The error of PMF estimation is ±1 kcal/mol, based on the spread of results calculated for shorter trajectories (6-8 and 8-10 ns).

Based on the comparison between the three force fields, it can be concluded that in combination with Berger lipids only the ECCR parameterization of ions is able to reproduce the binding of Ca^{2+} to phosphate groups of PC lipids which was reported in previous experimental

studies. Hence, the ECCR was chosen to be used in the remaining parts of the present study. Note that all force fields tested here indicate binding of Ca^{2+} to carbonyl groups of phospholipids and such a binding is also supported by the time-resolved fluorescence measurements performed here.

To further evaluate properties of the chosen force field combination, i.e., Berger lipids with ECRR scale-charge ions, we calculated the change of choline C-H bond order parameters in the so-called α and β segments (for definition, see Figure S1A) with increasing calcium concentration and compared them with experimentally measured ^{2}H NMR ΔνQ quadrupolar splitting taken from Altenbach and Seeling.[10] The results are presented in Fig. S8. With increasing concentration of calcium, the choline order parameters in MD simulations are reduced, qualitatively matching the experimental trends. Nevertheless, this reduction is quantitatively overestimated. Virtually the same overestimation was obtained for other currently available force fields (see the open collaboration research project nmrlipids.blogspot.fi that benchmarks available lipid force fields[11]). Such a quantitative discrepancy can be rationalized by overbinding of calcium by phospholipids. Nevertheless, the observed calcium binding sites predicted by the ECCR ion model, which are in agreement with our experiments, let us assume that the ECRR ions with Berger lipids can, at least semi-quantitatively, reproduce real calcium-phospholipid binding features.

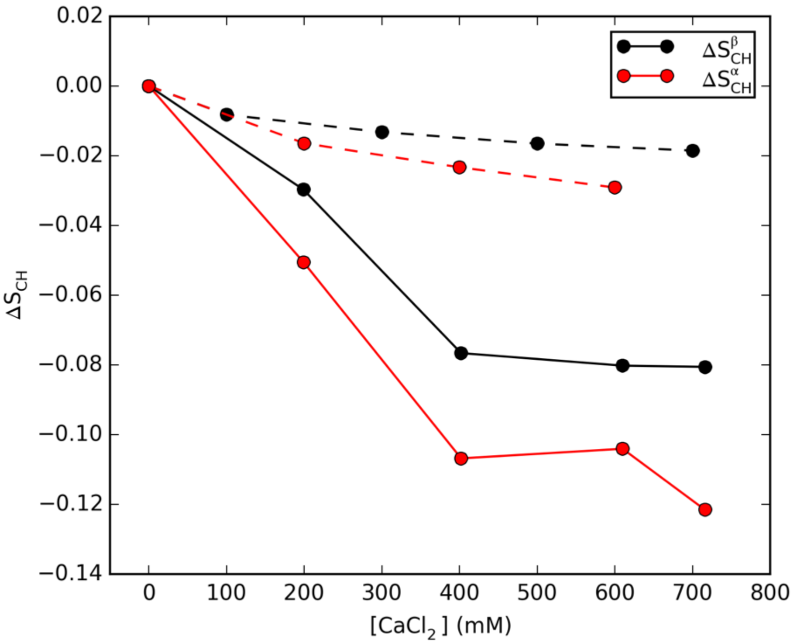

Fig. S8. Change of the order parameters in α and β segments of choline in POPC membranes as a function of $CaCl_2$ concentration in MD simulations (full lines) and NMR experiments[10] (dashed lines). Note that the experimental values for the β segment are available only for the $CaCl_2$ + 0.1 M NaCl system. The experimental values for the α segment are reported for purely $CaCl_2$ solutions.

8. MD – Charge Scaling and Water Models

The ECCR ionic parameters used here were recently derived based on 4 molar $CaCl_2$ solution in the SPC/E water.[5] In order to show that the parameters are transferable to SPC water used here as a default for Berger lipids, we repeated the simulations under the same conditions as in Ref. [5]. The mixed Lennard-Jones parameters were derived from self-parameters using Lorentz-Berthelot (LB) mixing rules and geometric averages (GA), respectively.
Besides the comparison with the neutron scattering data from Ref. 4, a comparison of the particle densities (Table S4), coordination numbers (Tables S4 and S5), peak positions of the radial distribution functions (Tables S5 and S6), and the self-diffusion coefficients (Table S7) with experimental values can be found in the following. The concentrations and temperatures in the experiments are the same as in the simulations unless stated otherwise. The neutron scattering experiments were performed in a previous study in heavy water with isotopically labeled calcium.[4] This approach makes it possible to evaluate the first order differences (for the exact definitions see Ref. 4 and the SI of Ref. [5]). The results of the first order difference functions both in r-space (ΔG(r)) and in the Q-space (ΔS(Q)) are shown in Fig. S9. Here, Q is the radial scalar part of the scattering vector. Both these functions contain the same information but they emphasize different structural aspects.

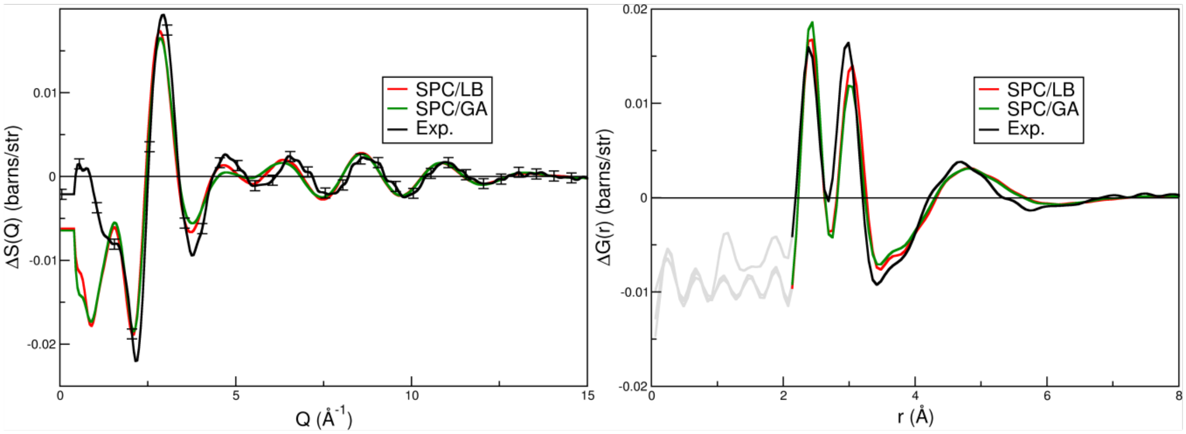

Fig. S9. First-order difference function in the Q-space (left-hand side) and the r-space (right-hand side) for the simulations with scaled charges in SPC water using Lorentz-Berthelot (LB) mixing rules in red and geometric averages (GA) in green. The experimentally measured data (in black) have an error of 0.0006 barns/str. For the sake of clarity, error bars are only shown for every 10th data point.

The first peak in the r-space representation is present mostly due to the oxygen atoms of the water molecules surrounding the calcium ions, the second one is caused by the corresponding hydrogen atoms. Note that the first peak also contains contributions from contact ion pairs with chloride (if present). The calculated functions in SPC water for both mixing rules correspond well with the experimental data in terms of peak positions and peak heights, although the comparison is slightly better for the calculations applying Lorentz-Berthelot mixing rules. The ratio between the first and the second peak is an indication for the amount of contact ion pairs in

the solution since there are two hydrogen atoms contributing to the second peak for each oxygen atom in the first solvation shell. This ratio is slightly worse for the calculations with the geometric average. This observation is in agreement with the first-shell coordination numbers for chloride around calcium (CN_{Cl}), as can be seen in Table S5.

Table S4. Comparison of the particle densities in SPC water models with Lennard-Jones parameters derived using Lorentz-Berthelot (LB) mixing rules and geometric averages (GA) with experimental values obtained from literature. Standard deviation < 0.0007 atoms/Å^3.

System	Particle density [atoms/Å^3]
SPC/LB	0.091
SPC/GA	0.092
Ref.[4]	0.096
Ref.[12]	0.094

Table S5. First-shell coordination numbers (CN_{H2O} and CN_{Cl}) and first- and second-shell RDF peak positions for calcium and chloride ($r_{1,2}$(Ca-Cl)) in SPC water models with Lennard-Jones parameters derived using LB and GA mixing rules.

System	CN_{H2O}	CN_{Cl}	r_1(Ca-Cl) [Å]	r_2(Ca-Cl) [Å]
SPC/LB	5.08 ± 0.02	0.99 ± 0.02	2.58 ± 0.01	3.9-5.7
SPC/GA	4.54 ± 0.02	1.49 ± 0.02	2.53 ± 0.01	4.0-5.8

Table S6. Sum of first-shell coordination numbers (CN_{H2O+Cl}) and first-shell RDF peak positions for calcium and oxygen (r_1(Ca-O)) employing SPC water models with Lennard-Jones parameters derived using LB and GA mixing rules including experimental results reported in the literature based on X-ray diffraction (XRD) and neutron scattering (ND).

System	CN_{H2O+Cl}	r_1(Ca-O) [Å]
SPC/LB	6.07 ± 0.04	2.37 ± 0.01
SPC/GA	6.03 ± 0.04	2.33 ± 0.01
Ref.[12] (XRD)	5.9 ± 0.3	2.45
Ref.[12] (ND)	6.5 ± 0.2	2.46 ± 0.02
Ref.[4] (XRD)[4]	6.8	2.43
Ref.[4] (ND)[4]	7.3	2.40 ± 0.01

Table S7. Self-diffusion coefficients of calcium (D^{Ca}) and chloride (D^{Cl}) calculated from the corresponding mean square displacement employing SPC water models with Lennard-Jones parameters derived using LB and GA mixing rules including experimental results reported in the literature

System	D^{Ca} [10^{-5} cm^2/s]	D^{Cl} [10^{-5} cm^2/s]
SPC/LB	0.37 ± 0.01	0.73 ± 0.05
SPC/GA	0.37 ± 0.03	0.63 ± 0.01
Ref.[13]	0.225 ± 0.002	0.447 ± 0.015

9. MD – Detailed Simulation Setup

Simulations were performed within the isothermal-isobaric (NpT) ensemble with temperature of 310 K controlled via the Nosé-Hoover algorithm with the coupling constant of 1 ps. A semi-isotropic barostat employing the Parinello-Rahman method with the coupling constant of 2 ps was used to control the pressure of 1.01 bar. The 3D periodic boundary conditions were employed. Short-range interactions were cut off at the distance of 1 nm. The Particle Mesh Ewald method with the grid spacing of 0.12 nm and the fourth order interpolation was employed to account for long-range electrostatic interactions.[14] The SETTLE and LINCS algorithms were employed for constraining the water molecules and bonds in lipids, respectively[15,16]. The time step of 2 fs was used for integration of equation of motions. Simulations were performed in the Gromacs software suite.[17] Visualization of trajectories was performed with the VMD code.[18]

10. References

1 Magarkar, A., Karakas, E., Stepniewski, M., Rog, T. & Bunker, A. Molecular Dynamics Simulation of PEGylated Bilayer Interacting with Salt Ions: A Model of the Liposome Surface in the Bloodstream. *Journal of Physical Chemistry B* **116**, 4212-4219 (2012).

2 Soong, R. & Macdonald, P. M. PEG molecular weight and lateral diffusion of PEG-ylated lipids in magnetically aligned bicelles. *Biochimica Et Biophysica Acta (BBA)-Biomembranes* **1768**, 1805-1814 (2007).

3 Berger, O., Edholm, O. & Jahnig, F. Molecular dynamics simulations of a fluid bilayer of dipalmitoylphosphatidylcholine at full hydration, constant pressure, and constant temperature. *Biophysical Journal* **72**, 2002-2013 (1997).

4 Badyal, Y. S., Barnes, A. C., Cuello, G. J. & Simonson, J. M. Understanding the Effects of Concentration on the Solvation Structure of Ca^{2+} in Aqueous Solution. II: Insights into Longer Range Order from Neutron Diffraction Isotope Substitution. *The Journal of Physical Chemistry A* **108**, 11819-11827 (2004).

5 Kohagen, M., Mason, P. E. & Jungwirth, P. Accurate description of calcium solvation in concentrated aqueous solutions. *The Journal of Physical Chemistry B* **118**, 7902-7909 (2014).

6 Kohagen, M., Lepsik, M. & Jungwirth, P. Calcium Binding to Calmodulin by Molecular Dynamics with Effective Polarization. *The Journal of Physical Chemistry Letters* **5**, 3964-3969 (2014).

7 Berendsen, H. J. C., Postma, J. P. M., Van Gunsteren, W. F. & Hermans, J. in *Intermolecular Forces* 331–342 (D. Reidel Publishing Company, 1981).

8 Bockmann, R. A. & Grubmuller, H. Multistep binding of divalent cations to phospholipid bilayers: a molecular dynamics study. *Angewandte Chemie* **43**, 1021-1024, (2004).

9 Jurkiewicz, P., Cwiklik, L., Vojtiskova, A., Jungwirth, P. & Hof, M. Structure, dynamics, and hydration of POPC/POPS bilayers suspended in NaCl, KCl, and CsCl solutions. *Biochimica Et Biophysica Acta-Biomembranes* **1818**, 609-616, doi:10.1016/j.bbamem.2011.11.033 (2012).

10 Altenbach, C. & Seelig, J. Calcium binding to phosphatidylcholine bilayers as studied by deuterium magnetic resonance. Evidence for the formation of a calcium complex with two phospholipid molecules. *Biochemistry* **23**, 3913-3920 (1984).

11 Miettinen, S. M. *et al.* NMRLipids project, http://nmrlipids.blogspot.fi/.

12 Megyes, T., Bakó, I., Bálint, S., Grósz, T. & Radnai, T. Ion pairing in aqueous calcium chloride solution: Molecular dynamics simulation and diffraction studies. *Journal of Molecular Liquids* **129**, 63-74 (2006).

13 Wang, J. H. Tracer-diffusion in Liquids. IV. Self-diffusion of Calcium Ion and Chloride Ion in Aqueous Calcium Chloride Solutions1. *Journal of the American Chemical Society* **75**, 1769-1770 (1953).

14 Essmann, U. *et al.* A smooth particle mesh Ewald method. *J Chem Phys* **103**, 8577-8593 (1995).

15 Hockney, R. W., Goel, S. P. & Eastwood, J. W. Quiet High-Resolution Computer Models of a Plasma. *Journal of Computational Physics* **14**, 148-158, (1974).

16 Hess, B., Bekker, H., Berendsen, H. J. C. & Fraaije, J. G. E. M. LINCS: A linear constraint solver for molecular simulations. *Journal of Computational Chemistry* **18**, 1463-1472, (1997).

17 Hess, B., Kutzner, C., van der Spoel, D. & Lindahl, E. GROMACS 4: Algorithms for Highly Efficient, Load-Balanced, and Scalable Molecular Simulation. *Journal of Chemical Theory and Computation* **4**, 435-447, (2008).

18 Humphrey, W., Dalke, A. & Schulten, K. VMD: visual molecular dynamics. *Journal of Molecular Graphics* **14**, 33-38, 27-38 (1996).
